# Direction-changeable lumbar cage versus traditional lumbar cage for treating lumbar spondylolisthesis: A retrospective study

**DOI:** 10.1097/MD.0000000000009984

**Published:** 2018-02-16

**Authors:** Haiping Zhang, Yonghong Jiang, Biao Wang, Qinpeng Zhao, Simin He, Dingjun Hao

**Affiliations:** a Department of Spine Surgery; b Department of Radiology, Honghui Hospital, Xi’an Jiaotong University Health Science Center, Xi’an, Shanxi, China.

**Keywords:** fusion, lumbar cage, lumbar spondylolisthesis, TLIF

## Abstract

Despite the diverse designs for the lumbar interbody fusion cage, there is no consensus on the optimal design to date. The current study aimed to compare the efficacy and complications associated with the direction-changeable and traditional lumbar cages for treating lumbar spondylolisthesis.

We conducted a retrospective study including 109 patients with lumbar spondylolisthesis, who were admitted to our hospital from January 2013 to December 2014. The patients were divided into the direction-changeable (group A) and traditional (group B) lumbar cage group.

All patients underwent single-level transforaminal lumbar interbody fusion and were followed up for 12 to 24 months. There were 52 cases in group A and 57 cases in group B. Surgery-related parameters, including operation time, bleeding volume, and hospitalization time, were recorded; there was no significant difference between the 2 groups regarding these parameters. The visual analog scale and Oswestry disability index at the last follow-up showed significant improvement compared with preoperative values in both groups (*P* *<* .05). Patients in group A demonstrated more intervertebral space height maintenance postoperatively than patients in group B but the difference was not statistically significant (*P* > .05). In group A, complications included 3 cases of nonunion (5.77%) and 1 case of cerebrospinal fluid leakage (1.92%). In group B, complications included 9 cases of nonunion (15.79%) and 1 case of postoperative infection (1.75%). There was a significant difference between both groups in terms of the nonunion rate and total complication rate (*P* < .05).

The direction-changeable lumbar cage has merits such as a higher bone fusion rate and fewer postoperative complications compared to the traditional lumbar cage.

## 1 Introduction

Surgical intervention has gained wide acceptance for treating lumbar spondylolisthesis. Transforaminal lumbar interbody fusion (TLIF) has a number of advantages, including less invasiveness and blood loss and a shorter hospital stay,^[1]^ and is becoming the standard technique for treating lumbar spondylolisthesis because of its efficacy and safety.^[2,3]^ Compared with posterior lumbar interbody fusion (PLIF), TLIF has merits such as the involvement of less nerve root retraction and a lower incidence of complications such as nerve root injury and spinal dura mater avulsion.^[4,5]^

A suitable interbody spacer or cage plays a pivotal role in successful bone fusion for both TLIF and PLIF. Different implants can lead to varied kinematic and mechanical changes and can affect fusion outcomes. Different spacer positions and shapes may not only markedly affect segmental lordosis but also alter neuroforaminal volume and area.^[6–8]^ Despite the diverse designs and materials for the interbody fusion cage, there is no consensus regarding the optimal choice. A bullet-shaped cage could achieve easy insertion and safe navigation around neural tissues, a biconvex cage could fit the concave shape between upper and lower endplates, while a cage with a serrated surface could stabilize the position of the interbody and avoid its migration.^[9]^ A favorable interbody cage can provide satisfactory axial support to prevent graft subsidence and limit postoperative segment mobility to facilitate graft fusion.

The direction-changeable lumbar cage is a kidney-shaped cage that emphasizes no dural retraction. It has been reported to achieve a high fusion rate and has gained wide acceptance.^[10]^ It can create and maintain sagittal lordosis while preventing subsidence and neurologic problems with the microscope-assisted placement of a structural allograft. In contrast, the traditional lumbar cage is a straight bullet-shaped cage that is placed obliquely through the disk space.^[11]^ In the current study, we retrospectively compared the effectiveness and complications associated with the direction-changeable and traditional lumbar cages in the treatment of lumbar spondylolisthesis, over an average of >20 months of follow-up.

## 2 Methods

### 2.1 Patient population

The inclusion criteria included patients: with radiography- and computed tomography (CT)-confirmed lumbar isthmic spondylolisthesis, with the lesion involving a single segment; and treated with the direction-changeable lumbar cage (group A) or traditional lumbar cage (group B) in our department from January 2013 to December 2014. The exclusion criteria were: previous history of lumbar spine surgery and insufficient radiographic follow-up data. This retrospective study was approved by the Institutional Review Broad of Honghui Hospital of Xi’an Jiaotong University, and all the patients provided signed informed consent.

### 2.2 Surgical procedures

**Figure 1:**
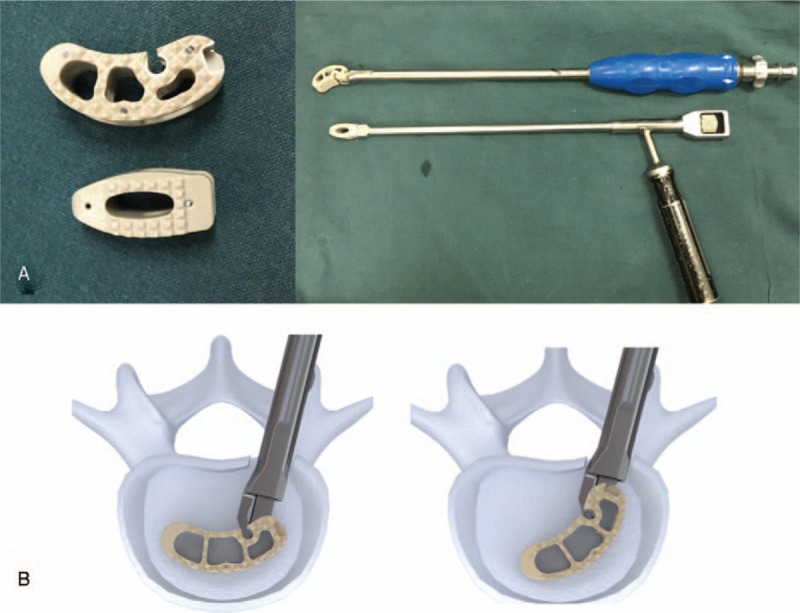
Direction-changeable and traditional cages (A). Direction-changeable cage, traditional cage, and device holder (B). The implantation process of the direction-changeable cage.

Surgical procedures for the enrolled patients were performed by the same group of surgeons. A median incision was made and a pedicle screw was placed, followed by spinal decompression and removal of the involved intervertebral disc. For group A, after implanting two-thirds of the cage, the cage-holding device was unscrewed to change the cage direction and to continue implanting the cage to the designated area (Fig. 1). The cage was placed at the anterior two-thirds of the vertebral body. For group B, the cage was placed inward and oblique to the intervertebral space. The cage could not exceed the anterior border of the vertebral body and was kept at least 3 mm away from the posterior border, and a position central to the intervertebral space was preferred.

### 2.3 Assessment parameters

Surgery-related parameters such as surgical duration, bleeding volume, and hospitalization time were collected. Preoperative and postoperative radiography (anterior-posterior and lateral view, as well as flexion and extension positions) and CT examinations were performed to assess the cage position and the degree of bone fusion. In addition, intervertebral space height was assessed with lateral radiography by measuring the distance between the endplate midpoints of the involved space. Bone fusion in our study was defined as: the nondetection of a radiolucent area between the cage and adjacent endplates on radiography, nondetection of motion at the fusion segment on a radiograph in the flexion and extension positions, and the presence of a bony bridge formation around the fusion segment. The visual analog scale (VAS), Oswestry disability index (ODI), and intervertebral space height were assessed before the procedure, 1 month postoperatively, and at the last follow-up. Complications, including nonunion and infection, were recorded.

### 2.4 Statistical methods

SPSS version 13.0 (IBM, Armonk, NY) was used for the statistical analysis. Discrete variables were compared using the chi-square or Fisher exact test. Continuous variables were compared using the Student *t* test. Statistical significance was set at *P* < .05.

## 3 Results

A total of 126 patients met the inclusion criteria; 14 cases with severe osteoporosis were excluded from this study. In group A, 3 cases were lost to follow-up and were further excluded. Thus, a total of 109 patients (67 male and 42 female) were included in the study: 52 patients in group A and 57 patients in group B. The average age in group A and B was 41 ± 5.4 and 44.3 ± 6.2 years, respectively (*P* > .05). The spondylolisthesis involved segments L3–L4, L4–L5, and L5–S1 in both groups. According to the Meyerding grading, the patients were all type II, type III, and type IV. The follow-up duration ranged from 12 to 24 months in both groups (*P* > .05). There were no significant differences in terms of baseline demographics, such as gender, age, and follow-up duration. The demographic details are listed in Table 1.

**Table 1:**
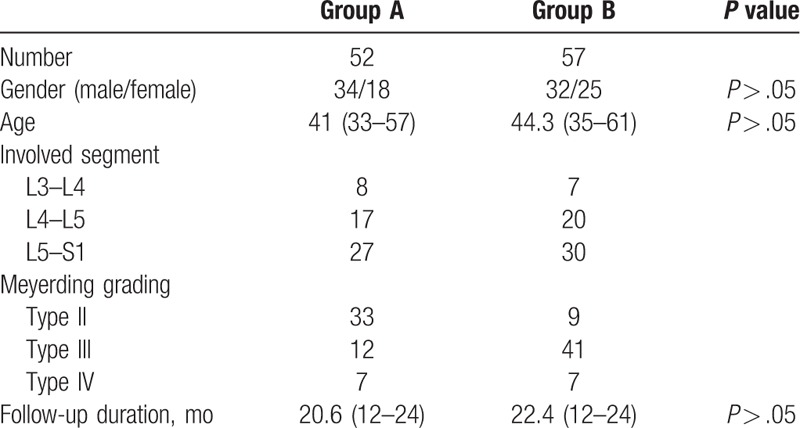
General patient information.

### 3.1 Surgery-related and postoperative parameters

The average surgery duration was 89.9 ± 14.4 and 87 ± 15.9 minutes in group A and B, respectively (*P* > .05). Bleeding volume was 156.6 ± 32.5 and 148.4 ± 44.6 mL in group A and B, respectively (*P* > .05). Hospitalization time was 10.1 ± 2.2 and 10.5 ± 3.1 days in group A and B, respectively (*P* > .05). The VAS and ODI at the last follow-up were 1.4 ± 0.7 and 11.9 ± 5.3, respectively, in group A and 1.3 ± 0.7 and 10.2 ± 6.9, respectively, in group B. Both parameters decreased significantly in both groups compared with preoperative values of 5.3 ± 1.7 and 35.1 ± 15.9, respectively, in group A and 5.2 ± 1.6 and 36.2 ± 10.3, respectively in group B (*P* < .05). However, there was no significant difference between the 2 groups with respect to VAS and ODI at the last follow-up (*P* > .05). Group A and B demonstrated an intervertebral space height of 8.9 ± 1.1 and 8.4 ± 1.2 mm, respectively, at the last follow-up (*P* > .05). No significant difference was found between the groups with respect to surgery-related parameters and postoperative parameters (Table 2).

**Table 2:**
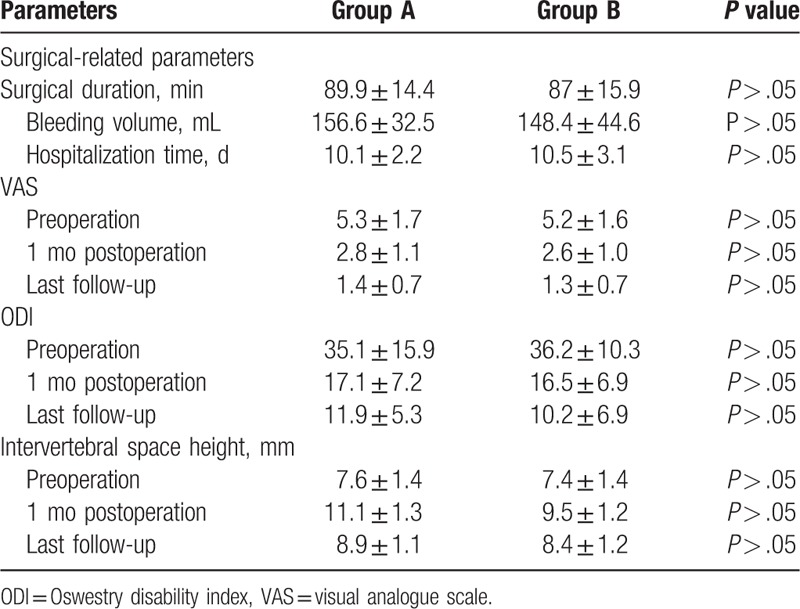
Surgical related and postoperative parameters.

### 3.2 Postoperative complications

**Figure 2:**
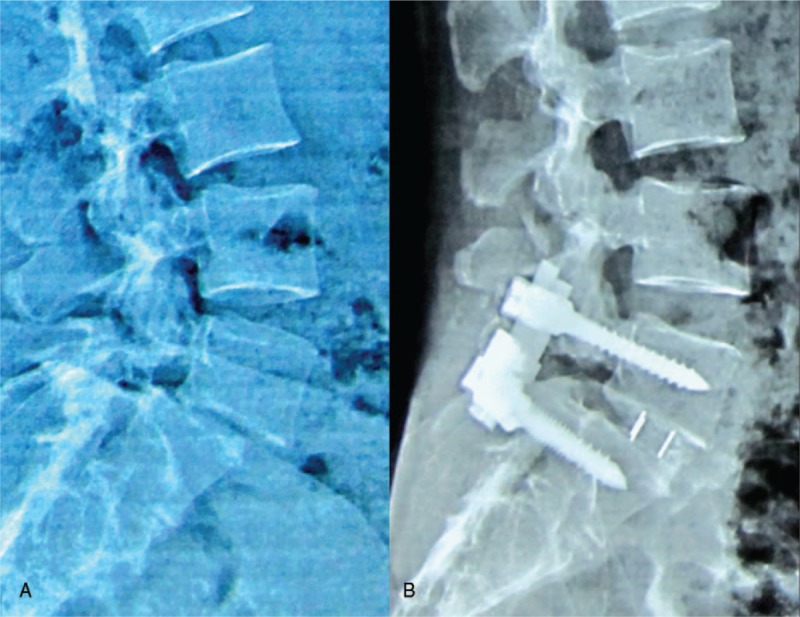
A case with direction-changeable cage (A). A 45-year-old male patient with spondylolisthesis at L4-5 (B). After the implantation of the direction-changeable cage, the intervertebral space height and fusion segment lordotic angle were restored.

**Figure 3:**
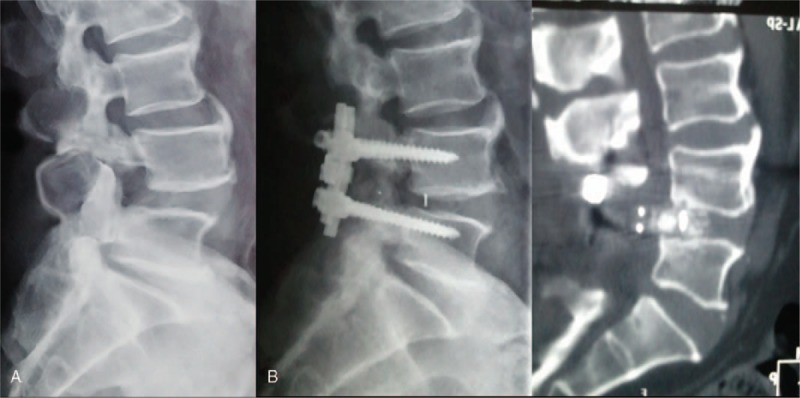
A case with traditional cage (A). A 52-year-old male patient with spondylolisthesis at L4-5 (B). Traditional cage retrocession 2 months after the implantation combined with right limb symptoms.

In group A, there were 3 cases (5.77%) of interbody nonunion and 1 case (1.92%) of cerebrospinal fluid leakage during cage implantation. They were treated with delayed extubation and showed great improvement after conservative treatment. In group B, there were 9 cases (15.79%) of interbody nonunion, among which 5 cases (8.78%) exhibited cage retrocession, 4 underwent revision surgery and 1 case without neurologic symptoms did not receive further treatment. Moreover, 1 case (1.75%) of cage subsidence also received conservative treatment because no neurologic deficit was observed. In addition, developed postoperative infection developed in 1 case (1.75%) and was treated with antibiotics. There was a significant difference in the total postoperative complication rate between group A (7.69%) and group B (17.54%) (*P* < .05) (Table 3). Radiological information regarding the 2 cages is listed in Figs. 2 and 3.

**Table 3:**
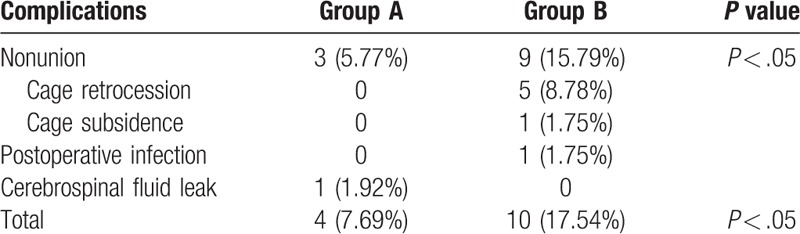
Postoperative complications.

## 4 Discussion

In recent years, lumbar interbody fusion has gained wide acceptance for the treatment of lumbar spondylolisthesis, as it can maintain not only the physiological curvature of the spine and the original intervertebral space height but also bear spinal axial loading. TLIF has certain merits, including a shorter operative time, less blood loss, shorter hospital stay, and lower complication rates, compared with other surgical procedures such as anterior lumbar interbody fusion/PLIF.^[1]^ The current study was retrospective and compared the treatment efficacy between 2 TLIF implants, namely, the direction-changeable and traditional lumbar cages for treating lumbar spondylolisthesis.

Satisfactory bone fusion can effectively prevent postoperative cage shifting and postoperative intervertebral space height loss.^[12,13]^ Intervertebral cages have the merits of increasing the foraminal height and cross-sectional area during fusion procedures.^[14]^ The shape of the direction-changeable lumbar cage applied here is similar to that of the renal cage reported by Yang et al,^[15]^ which has a larger grafting bone volume than the traditional lumbar cage. Prolo et al^[16]^ suggested that successful bone fusion is mainly determined by effective grafting bone contact area, such cages applied in anterior lumbar laminectomy and fusion could correct kyphosis by 11.8° ± 7.1° and achieve a bone fusion rate of 90.2%. In our study, there were 3 cases of bone nonunion and 1 case of cerebrospinal fluid leakage in the direction-changeable cage group with a bone fusion rate of 94.5%, which is consistent with the Yang et al study.^[15]^ For the traditional lumbar cage, extra bone grafting around the cage could also increase the effective grafting bone contact area. However, according to our results, group B had more cases of interbody nonunion compared to group A. Graft fusion could be influenced by many factors, and it has been speculated that stress shielding and/or limited vascular penetration might inhibit bone graft fusion in the interior part of cages.^[17]^ In the early stage of inflammatory reaction and the late stage of callus remodeling of bone grafting, along with osteoblasts participating in bridging callus formation, osteoclasts could absorb necrotic bone and bone tissue outside the stress axis.^[18]^ Stress shielding and uneven endplate stress exist in traditional lumbar cages, suggesting that the 9 cases with nonunion in the traditional lumbar cage group might have been due to grafting bone particle absorption.

Compared to group B, we observed no cases of vertebral subsidence and less intervertebral space height loss in group A, which is consistent with a previous study by Rice et al.^[11]^ Improved intervertebral disc space height cannot only alleviate kyphosis but also improve modified Odom criteria scores and prevent neurological symptoms such as leg pain.^[19]^ As the compression force applied during pedicle screw tightening could cause minimal cage migration, patients with poor bone quality are more prone to experience cage subsidence. A sufficient amount of cage-endplate contact stress under compression is critical for cage placement maintenance. The use of a direction-changeable lumbar cage can yield a small and balanced stress distribution, which could prevent postoperative cage subsidence.^[20]^ In addition, the position of cage placement is also an important factor for the prevention of cage subsidence. A direction-changeable lumbar cage is usually placed on the anterior side of the disc. In contrast, the traditional lumbar cage is usually placed lateral to the disc. Fukuta et al^[14]^ reported that a renal cage could markedly decrease the vertebral subsidence rate when placed at the anterior part of the vertebral body. In addition, Kepler et al^[19]^ reported that subsidence rates of 14.1% (19 of 135) and 1.9% (2 of 103) were associated with 18- and 22-mm-wide cages, respectively, indicating that wider intervertebral cages could yield a significantly lower rate of subsidence. We suggest that anterior placement could provide better support for the anterior column and maintain the lordotic angle. Additionally, another study by Fukuta et al^[14]^ suggested that kidney-type cages should be located in the anterior portion of the intervertebral space to prevent subsidence of the intervertebral body, especially in elderly patients.

Cage migration is another important complication in TLIF, and malpositioned cages could cause severe adverse effects. We observed 5 cases of case retrocession in group B, while no case was observed in group A. Patients often complain of exacerbated neurological symptoms because posterior migration can cause the compression of nerve roots on the dura mater, and revision surgeries are often necessary. In line with our observation, kidney-shaped and large cages have been reported to be more stable and have a lower incidence of cage migration compared with rectangular and small cages.^[21]^ In addition to the shape of implants, other factors such as implant number, size, and implantation site should not be overlooked.^[20,22]^ Moreover, the cage material could also influence the fusion outcomes. Smith et al^[23]^ reported that poly-L/DL-lactide implants migrated more frequently than carbon fiber implants. The above factors should be taken into account for the future development of favorable intervertebral implants.

There are several limitations to the current study. Because it was a single-center study with a limited sample size, a selection bias could not be excluded. Moreover, we did not discuss cage efficacy in terms of different grades of spondylolisthesis. A larger sample size is needed to draw a more statistically relevant conclusion. Furthermore, bone mineral density may have varied among enrolled patients and could also have affected bone fusion rate and time. In addition, a longer follow-up duration could have yielded a more comprehensive conclusion in terms of postoperative complications and bone fusion rate. Further studies are needed to clarify the issues mentioned above.

In conclusion, the direction-changeable lumbar cage is advantageous compared to the traditional lumbar cage in terms of a higher bone fusion rate and fewer postoperative complications, such as cage retrocession. However, both implants could yield satisfactory treatment efficacy.

## References

[1] Hee HT Castro FP Jr Majd ME Anterior/posterior lumbar fusion versus transforaminal lumbar interbody fusion: analysis of complications and predictive factors. J Spinal Disord 2001;14:533–40. 11723406 10.1097/00002517-200112000-00013

[2] Taneichi H Suda K Kajino T Unilateral transforaminal lumbar interbody fusion and bilateral anterior-column fixation with two Brantigan I/F cages per level: clinical outcomes during a minimum 2-year follow-up period. J Neurosurg Spine 2006;4:198–205. 16572618 10.3171/spi.2006.4.3.198

[3] Holly LT Schwender JD Rouben DP Minimally invasive transforaminal lumbar interbody fusion: indications, technique, and complications. Neurosurg Focus 2006;20:E6. 10.3171/foc.2006.20.3.7 16599422

[4] Humphreys SC Hodges SD Patwardhan AG Comparison of posterior and transforaminal approaches to lumbar interbody fusion. Spine (Phila Pa 1976) 2001;26:567–71. 11242386 10.1097/00007632-200103010-00023

[5] Cole CD McCall TD Schmidt MH Comparison of low back fusion techniques: transforaminal lumbar interbody fusion (TLIF) or posterior lumbar interbody fusion (PLIF) approaches. Curr Rev Musculoskelet Med 2009;2:118–26. 19468868 10.1007/s12178-009-9053-8 PMC2697340

[6] Godde S Fritsch E Dienst M Influence of cage geometry on sagittal alignment in instrumented posterior lumbar interbody fusion. Spine 2003;28:1693–9. 12897494 10.1097/01.BRS.0000083167.78853.D5

[7] Groth AT Kuklo TR Klemme WR Comparison of sagittal contour and posterior disc height following interbody fusion: threaded cylindrical cages versus structural allograft versus vertical cages. J Spinal Disord Tech 2005;18:332–6. 16021014 10.1097/01.bsd.0000163037.17634.89

[8] Chen D Fay LA Lok J Increasing neuroforaminal volume by anterior interbody distraction in degenerative lumbar spine. Spine (Phila Pa 1976) 1995;20:74–9. 7709283 10.1097/00007632-199501000-00014

[9] Cho W Wu C Mehbod AA Comparison of cage designs for transforaminal lumbar interbody fusion: a biomechanical study. Clin Biomech 2008;23:979–85. 10.1016/j.clinbiomech.2008.02.008 18675496

[10] Anand N Hamilton JF Perri B Cantilever TLIF with structural allograft and RhBMP2 for correction and maintenance of segmental sagittal lordosis: long-term clinical, radiographic, and functional outcome. Spine (Phila Pa 1976) 2006;31:E748–53. 16985443 10.1097/01.brs.0000240211.23617.ae

[11] Rice JW Sedney CL Daffner SD Improvement of segmental lordosis in transforaminal lumbar interbody fusion: a comparison of two techniques. Global Spine J 2016;6:229–33. 27099813 10.1055/s-0035-1559583 PMC4836934

[12] Chen L Yang H Tang T Cage migration in spondylolisthesis treated with posterior lumbar interbody fusion using BAK cages. Spine (Phila Pa 1976) 2005;30:2171–5. 16205342 10.1097/01.brs.0000180402.50500.5b

[13] McAfee PC DeVine JG Chaput CD The indications for interbody fusion cages in the treatment of spondylolisthesis: analysis of 120 cases. Spine (Phila Pa 1976) 2005;30 (6 Suppl):S60–5. 10.1097/01.brs.0000155578.62680.dd 15767888

[14] Fukuta S Miyamoto K Hosoe H Kidney-type intervertebral spacers should be located anteriorly in cantilever transforaminal lumbar interbody fusion: analyses of risk factors for spacer subsidence for a minimum of 2 years. J Spinal Disord Tech 2011;24:189–95. 20634726 10.1097/BSD.0b013e3181e9f249

[15] Yang X Song Y Liu L Anterior reconstruction with nano-hydroxyapatite/polyamide-66 cage after thoracic and lumbar corpectomy. Orthopedics 2012;35:e66–73. 22229617 10.3928/01477447-20111122-10

[16] Prolo DJ Oklund SA Butcher M Toward uniformity in evaluating results of lumbar spine operations. A paradigm applied to posterior lumbar interbody fusions. Spine 1986;11:601–6. 3787326 10.1097/00007632-198607000-00012

[17] Steffen T Tsantrizos A Fruth I Cages: designs and concepts. Eur Spine J 2000;9 suppl 1:S89–94. 10766063 10.1007/PL00010027 PMC3611434

[18] Togawa D Bauer TW Brantigan JW Bone graft incorporation in radiographically successful human intervertebral body fusion cages. Spine (Phila Pa 1976) 2001;26:2744–50. 11740367 10.1097/00007632-200112150-00025

[19] Kepler CK Rihn JA Radcliff KE Restoration of lordosis and disk height after single-level transforaminal lumbar interbody fusion. Orthop Surg 2012;4:15–20. 22290814 10.1111/j.1757-7861.2011.00165.x PMC6583197

[20] Chen SH Lin SC Tsai WC Biomechanical comparison of unilateral and bilateral pedicle screws fixation for transforaminal lumbar interbody fusion after decompressive surgery–a finite element analysis. BMC Musculoskelet Disord 2012;13:72. 22591664 10.1186/1471-2474-13-72 PMC3503692

[21] Zhao FD Yang W Shan Z Cage migration after transforaminal lumbar interbody fusion and factors related to it. Orthop Surg 2012;4:227–32. 23109307 10.1111/os.12004 PMC6583644

[22] Comer GC Behn A Ravi S A biomechanical comparison of shape design and positioning of transforaminal lumbar interbody fusion cages. Global Spine J 2016;6:432–8. 27433426 10.1055/s-0035-1564568 PMC4947403

[23] Smith AJ Arginteanu M Moore F Increased incidence of cage migration and nonunion in instrumented transforaminal lumbar interbody fusion with bioabsorbable cages. J Neurosurg Spine 2010;13:388–93. 20809735 10.3171/2010.3.SPINE09587

